# Measuring and Modeling Mechanical Ventilation for Long-Term Environmental Monitoring in Large Commercial Laying Hen House

**DOI:** 10.3390/ani14223339

**Published:** 2024-11-20

**Authors:** Ji-Qin Ni

**Affiliations:** Department of Agricultural and Biological Engineering, Purdue University, 225 South University Street, West Lafayette, IN 47907, USA; jiqin@purdue.edu

**Keywords:** animal environment, animal system management, building ventilation, indoor climate, poultry building, ventilation measurement, weather impact

## Abstract

Determining ventilation rates in commercial animal buildings has been technically challenging. This study aimed to develop an innovative method and a ventilation model and provide new insights into animal building ventilation. A layer house with 46 fans was studied over six months. A full-size and fast-response portable fan tester was developed for on-site fan ventilation measurement. Results indicated that the house differential pressures (dP) varied from +10.4 to <−100.0 Pa but remained between −10 and −30 Pa for 75.7% of the time. The mean house dP was −18.1 ± 8.9 Pa (mean ± standard deviation). Fan rotational speeds ranged from 495 to 580 rpm, with an average of 555 ± 14 rpm. Daily mean house ventilation rates ranged from 1800 to 22,142 m^3^ min^−1^, averaging 4.68 m^3^ h^−1^ per hen. This study concluded that house dP can be greatly affected by strong winds in addition to fan operations and air inlet openings. Fan rotational speeds are influenced by pulley sizes and fan belt maintenance. On-site fan tests with the fan tester can generate reliable data for fan ventilation characterization. Fan models that incorporate both fan rotational speeds and differential pressures considerably improve ventilation rate calculations.

## Simple Summary

Ventilation is essential for keeping animals comfortable in buildings and controlling air pollutant emissions. However, accurately measuring ventilation rates in large commercial animal facilities is challenging. The purpose of this research was to improve our understanding of ventilation in such buildings and to develop a new method for calculating ventilation rates using new technology. This study was conducted in a large commercial laying hen house with 46 fans in the Midwestern U.S. over six months, covering both cold and hot seasons. An innovative portable fan tester was designed to measure the fan performance and to develop a ventilation model. Results showed that the air pressure inside the house was influenced by external winds and fan operations. The house ventilation rates varied seasonally, with an average of 4.68 cubic meters per hour per hen. This research demonstrated that combining fan speed and air pressure data considerably improved ventilation rate calculations. The portable fan tester provided reliable on-site measurements, making ventilation rate assessments more accurate.

## 1. Introduction

The United Nations recently updated the predicted world population, projecting growth to be around 8.5 billion by 2030 and 9.7 billion by 2050 [1]. The surge in population continues to boost food demand and, consequently, food animal production. Modern animal agriculture has significantly enhanced the efficiency of meat, egg, milk, and fiber production. However, alongside these advancements, the development of animal agriculture has raised environmental concerns, particularly regarding indoor air quality in animal buildings, and pollutant emissions from these structures into the outdoor atmosphere, e.g., [2]. Major air pollutants in and emitted from animal agriculture include particulate matter (PM), ammonia (NH_3_), hydrogen sulfide (H_2_S), greenhouse gases, and odor.

Ventilation plays a critical role in maintaining a conducive environment within animal buildings and other indoor environments, e.g., [3]. It regulates indoor temperature, moisture levels, and pollutant concentrations, and is, therefore, directly related to heat stress, e.g., [4], and indoor air quality. This, in turn, affects the safety and health of the workers, e.g., [5], and the safety, health, productivity, and behavior of the animals [6].

Ventilation rates are pivotal for calculating air pollutant emissions because the emission rate is the product of the ventilation rate and the pollutant concentration difference between the air inlet and outlet of the building. Therefore, unreliable ventilation measurements in animal buildings could introduce considerable errors into scientific research and environmental regulations.

Building ventilation rates can be affected by many factors. Measuring ventilation rates in naturally ventilated animal buildings poses more technical challenges and greater uncertainties compared with mechanically ventilated buildings. Nevertheless, reliably measuring ventilation rates in mechanically ventilated animal buildings also remains a major challenge, particularly in buildings equipped with many large (e.g., >1-m diameter) wall fans.

Wall fans in mechanically ventilated animal buildings are sensitive to the differential pressures (dP) that the fans must overcome [7]. Differential pressures in these buildings are influenced by wind velocity, fan operational status, and air inlet opening. Ventilation at commercial animal buildings is subject to various weather conditions and building management practices. Studying the impact of natural condition variations on ventilation requires long-term research covering both cold and hot seasons. Some investigations reported dP in laying hen houses, e.g., average at −36.5 Pa in one house and −48.9 Pa in another, during two years of field monitoring [8]. However, comprehensive studies on dP in commercial poultry houses, especially those affected by weather conditions, are still lacking.

Various ventilation monitoring methods have been developed and tested in mechanically ventilated animal buildings over the past decades. These methods employ either indirect approaches, providing measurement data in alternative units that necessitate post-measurement calculations and conversions to volume per unit time, or direct approaches, offering measurement data directly in volume per unit time [9].

For direct measurement, a ventilation sensor for a building exhaust chimney with a diameter of 0.5 m was developed in Belgium in 1983 [10]. This sensor was designed for long-term measurement and installed at the inlet or upstream of the ventilation fan. It offered highly reliable ventilation rate measurements in livestock building studies across Europe, e.g., [11,12]. However, it is unsuitable for livestock and poultry buildings with larger wall fans, such as those with diameters of 1.2 or 1.5 m in North America.

Another type of sensor reported for ventilation measurement in poultry buildings was the IRIS damper (Continental Fan Manufacturing Inc., Buffalo, NY, USA). These devices measure pressure drops caused by airflow through a duct and convert the pressure drops to airflow rates. The dust size is limited to 81 cm. So far, the only reported use of this device in laying hen housing was the 204 mm IRIS damper in a bench-scale system in Canada [13].

To measure the ventilation rate of large fans, a portable device consisting of a vertically moving array of five impeller anemometers was developed in the U.S. for sporadic fan testing. These anemometers traveled from the top to the bottom of a large fan’s inlet or exhaust opening to complete one measurement in less than 4 min [14]. An improved model, called FANS (fan assessment numeration system), demonstrated excellent performance in laboratory calibration [15]. It was widely used in U.S. air quality studies in animal agriculture, e.g., [16,17,18]. However, it was susceptible to errors in field conditions because of its slow response in minutes compared with the fast-varying dP, which can be within seconds. Its large size and requirement of AC power or a generator also made it impractical to apply in certain field situations [16], necessitating the development of new methods.

The application of data driven methods have been adopted in building environment studies, e.g., [19], and in animal farming, e.g., [20]. These methods (such as data mining, machine learning, and artificial intelligence) depend heavily on data from the real world for model training and accuracy improvement. However, the availability of long-term measurement data from commercial animal buildings is still very limited. Therefore, we need more reliable and real-world data and better understanding of the data we have.

A six-month field study was conducted in a large commercial laying hen house to determine particulate matter emissions and generate new building environment data. A comprehensive measurement approach and a high-level quality assurance and quality control plan were adopted in this study. The objective of this paper was to demonstrate the new research and the final findings of the house ventilation study. Specifically, this work explored (1) methodology for multi-method ventilation monitoring, including an innovative full-size portable fan tester; (2) characteristics of house dP, fan rotational speeds, house ventilation rates under different weather, and field operational conditions; and (3) an improved model to calculate fan ventilation rates based on both dP and fan rotational speeds.

## 2. Materials and Methods

### 2.1. Experimental Site

#### 2.1.1. Laying Hen House

**Figure 1:**
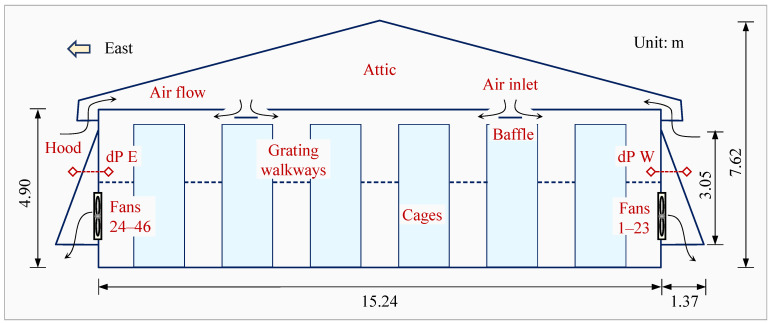
Schematic of the cross-sectional north end view of the 152.40 m long two-story laying hen house. dP E and dP W are paired differential pressure measurement ports across east sidewall and west sidewall, respectively.

This research took place in a two-story commercial manure belt laying hen house on an egg farm in Midwestern U.S., where this comprehensive air quality study was conducted from 15 March to 15 September 2021, spanning cold and hot seasons. The house was oriented north to south and measured 15.24 m width × 152.40 m length × 7.62 m ridge height (4.90 m sidewall height). The two stories were separated by metal grating walkways along the cage rows (Figure 1). During the study period, the house raised around 140,000 W36 Hyline laying hens in six rows and seven tiers of cages. The average weight of birds in the house was 1.50 ± 0.05 kg (mean ± standard deviation). At the start of this study, the birds were 36 weeks old. An artificial molting commenced on 12 July and concluded on 2 September.

#### 2.1.2. House Ventilation System

The house was mechanically ventilated and negatively pressurized. It had six slotted ceiling baffle style air inlets, measuring 0.42 m in width and with a maximum drop of 0.22 m, arranged in two rows and three sections along the house. These inlets were wire- and motor-controlled using two AEI control systems (AEI, Renville, MN, USA) based on dP and temperature in the house.

House ventilation was provided by 46 single-speed exhaust ventilation fans, with half installed in the west sidewall (fans 1–23) and the other half in the east sidewall (fans 24–46, Figure 1). These 46 fans were controlled across 12 fan stages based on house temperatures, with each stage comprising 2–6 fans (Table 1). Fans on the west wall were controlled by odd-numbered stages, and those on the east wall were controlled by even-numbered stages. Stages 9 and 10 consisted of cycling fans that were frequently turned on and off.

#### 2.1.3. Ventilation Fans

The fans were 1.12 m diameter RayDot fans (RayDot, Cokato, MN, USA) with 1.32 m × 1.32 m fan boxes. These 3-blade fans were belt-driven using 3-phase 0.746 kW (1-hp) motors. The space between the fans was 3.73 m. The fans had no exhaust discharge cones installed but had identical exhaust hoods outside the sidewalls (Figure 1). Each hood measured 3.05 m in height and 1.60 m in width and had a bottom depth of 1.37 m (with a top depth of 0.00 m), providing a bottom opening area of 2.192 m^2^ for air exhaust.

The Bioenvironmental and Structural Systems (BESS) Lab at the University of Illinois Urbana-Champaign published test results for some RayDot fans [21]. The closest tested fan to those installed in the house was a 1.12 m diameter RayDot PMC-4821BD fan (fan test #97323). This fan was belt-driven by a 0.746 kW Magnetek 8-182505 motor (Magnetek, Inc., Menomonee Falls, WI, USA) and had a fiberglass housing, plastic shutter, guard, and a discharge cone. Although the test conditions differed from those of the installed fans in the house, the test results served as a good reference for understanding the fan performance at various dP and rotational speeds (Table 2).

#### 2.1.4. Mobile Lab and On-Site Computer System

A 7.32 m length × 2.43 m width × 2.74 m height mobile lab was set up 10 m west of the house for this study. This setup followed the protocol in some large air quality monitoring campaigns in the U.S., such as the Aerial Pollutant Emissions from Animal Confinement Buildings [22] and the National Air Emission Monitoring Study [23]. The mobile lab housed a data acquisition system that was connected to 67 different measurement instruments and sensors. All these instruments and sensors were hardwired to the system to ensure reliability, except for a weather station, which was connected wirelessly.

An on-site computer system collected analog and digital outputs from all the instruments and sensors at 1 Hz, converted the signals to engineering units, and computed averages over 15 s and 1 min intervals. These mean values were saved in two separate data files on the computer, which also performed automatic daily data pre-processing [24]. The desktop computer was remotely accessed for real-time monitoring of the data displayed in AirDAC software (version 4.2).

### 2.2. Continuous and Real-Time Ventilation Monitoring

#### 2.2.1. Fan Rotational Speed Measurement

The rotational speeds of each of the 46 fans were continuously measured using a normally open NPN Hall effect sensor (Model NJK-5002C, made in Taiwan) paired with two magnets. The sensor, with a diameter of 12 mm and an overall length of 37 mm, has an effective detection distance of 0–10 mm and operates within a supply voltage range of 5–30 VDC. The sensor was attached to the fan motor frame using a bracket.

The two magnets were symmetrically positioned on opposite sides of the fan blade pulley to balance the weight of the pulley. As the pulley rotated, each magnet passed by the Hall effect sensor sequentially, triggering two pulse signals per 360° rotation of the pulley. These pulse signals were recorded as counts per minute, divided by two, and converted into fan blade rotation speeds (rpms) in AirDAC.

#### 2.2.2. Differential Pressure Measurement

Differential pressures across the west and east walls were continuously monitored using two multi-range differential pressure transducers (Model 260, Setra Systems Inc., Boxborough, MA, USA) following the Quality Assurance Project Plan of the National Air Emission Monitoring Study [25]. These transducers, with an accuracy of ±0.5 Pa, were set to a measurement range of ±100 Pa and an output signal range of 0–10 VDC. They were shunted to calibrate zero and verified with an inclined manometer at various span pressures. Though the transducer specifications did not list response times, observations indicated that the displayed digital data updated in less than one sec. The transducers were installed inside the mobile lab, with their ports extended to the pressure measurement locations using 0.48 cm inside diameter PVC tubing.

#### 2.2.3. Weather Measurement

A Davis 6152 Wireless Vantage Pro2 weather station (Davis Instruments, Hayward, CA, USA) on a mounting tripod was set up approximately 10 m above ground on the rooftop of an adjacent building. It measured wind direction and speed, ambient temperature, relative humidity, solar radiation, and rainfall. The collected data were wirelessly transmitted in real-time to a Vantage Pro2 Console (Davis Instruments) located in the mobile lab and connected to the data acquisition and control computer. Data updates occurred every minute, and timestamps were synchronized with all online measurement variables. Wind data were used to assess the impact of wind velocity on house pressures and ventilation. The actual resolution of wind speed in the recorded data every min was 1.6 km h^−1^, although the manufacturer-specified wind speed resolution of the weather station was 1 km h^−1^.

### 2.3. Development of a Portable Fan Tester

#### 2.3.1. Full-Size Portable Fan Tester

A full-size portable fan tester (PFT) was designed and developed to evaluate the performance of individual ventilation fans under varying dP and rotational speeds. The PFT was based on the traverse principle, endorsed by the Air Movement and Control Association International [26], and the wall fan tester (WFT) for wall fans of 0.356 m and 0.508 m diameters [9]. It was designed for large ventilation fans in commercial animal buildings, with full portability.

The PFT was composed of a lightweight frame of 0.203 m depth and a 1.335 m × 1.335 m opening area (1.7822 m^2^). A total of 5 vertical aluminum bars supported 5 × 5 (total of 25) equally spaced impeller anemometers (Model 27106T, RM Young, Traverse City, MI, USA). Each anemometer had a 20 cm diameter 4-blade carbon fiber thermoplastic impeller (Part number 08254, RM Young). The anemometer had a threshold of 0.4 m s^−1^ and a measurement range of 0–35 m s^−1^ [27]. The 5 × 5 traverse method was also applied to large fan air speed measurement using hot wire anemometers in laying hen houses in Europe [28,29].

Each of the 25 opening sub-areas in the PFT was 0.0713 m^2^. The circular area covered by a freely rotating anemometer impeller was 0.0314 m^2^, covering 44% of the sub-area. The mean air speed in the sub-area was represented by that in the anemometer covering area, as in the traverse method [26].

Two steel hanging hooks were fixed at the top of the PFT for easy placement of the PFT on a fan for ventilation measurement. The surface of the wooden frame facing the fan inlet was covered with a 2.0 cm wide and 1.3 cm thick self-adhesive soft foam weatherstrip to prevent air leakage during fan testing. Mobility was facilitated by four caster wheels, allowing the PFT to be easily moved between different fans.

#### 2.3.2. Data Acquisition of the PFT

The 25 anemometers were connected to a data acquisition device (Model USB-1624, Measurement Computing Co., Norton, MA, USA) that provided 32 single-ended analog input channels at a 24-bit resolution. Powered by a 5-VDC battery power bank, the device was connected to a laptop via a USB cable. The laptop, running AirDAC, acquired data at 1 Hz and recorded average values every 15 s and 60 s in two parallel files. These data were timestamped and included 25 averaged voltage output variables from the anemometers. Time synchronization between the laptop and another desktop in the mobile lab ensured consistency across data recording systems.

#### 2.3.3. Calibration of Anemometers

Calibration was performed in individual anemometers as the PFT was based on the traverse principle [26]. Prior to using the PFT for fan ventilation measurement in the house, all the anemometers went through recalibration to check the bearing torque and test the generator output, as required in USEPA [30]. The anemometer bearing torque was checked with the anemometer torque disc (part number 18310, RM Young) [31]. The anemometer generator outputs were calibrated with the selectable speed anemometer drive (Model 18802, RM Young) at 8 rotational speeds from 0 to 2100 rpm at 300 rpm increments. The anemometer voltage outputs were measured with a multimeter (Model 189, Fluke Corporation. Everett, WA, USA) that had a 50,000-count resolution and a 0.025% basic DC accuracy.

#### 2.3.4. Calculation of Airflow Through the PFT

The method used to calculate airflow through the PFT was based on the PFT’s design and the specifications of the anemometers, as shown in Equation (1).

(1) $$Q_{PFT}\;=\;\;\sum_{i=1}^{25}S\cdot W_{i}$$

where *Q_PFT_* is the airflow rate through the PFT, m^3^ min^−1^; *S* is the opening sub-area, 0.0713 m^2^; and *W_i_* is the air speed measured with the *i*-th anemometer, m min^−1^.

*W_i_* was calculated with Equation (2).

(2) $$W_{i}\;=\;\;PT\cdot K_{i}\cdot V_{i}$$

where *PT* is the pitch of the impeller, 0.300 m revolution^−1^; *K_i_* is the coefficient related to the *i*-th anemometer, revolution min^−1^ VDC^−1^, obtained during the anemometer calibration; and *V_i_* is the voltage output of the *i*-th anemometer, VDC.

### 2.4. Fan Characterization and House Ventilation Determination

#### 2.4.1. On-Site Fan Testing

Fan testing was conducted on-site for each of the 46 fans using the PFT to characterize the fan airflow rates from 4 to 6 May. To minimize the interference with the normal house operation, most fan airflow measurements were carried out under existing ventilation control conditions. If a fan being tested was not operating, it was manually turned on for the duration of its test and then reverted to its automatic setting. The measurement of each fan ranged from 4 to 12 min, during which wall dP was monitored in real time to determine the appropriate measurement duration. Durations were selected to capture stabilized dP, lasting at least one min.

#### 2.4.2. Data Processing and Fan Model Development

Two files from two computers containing 15 s averaged data were merged using synchronized timestamps. One file was from the laptop recording the PFT outputs. Another file was from the desktop capturing dP, fan rotational speeds, and other measurement variables. The data during the tests were cross-referenced with the field test logs that detailed each testing operation step by step. Valid data segments consisting of at least 1 min of 4 consecutive 15 s records were selected based on relatively stable dP (<±3%) during that time. Fan ventilation rates during that time were correlated with the dP and fan rotational speeds in the two data files and averaged. This process generated multiple valid data subsets that were used to develop a fan model of fan ventilation rates as functions of dP and fan rotational speeds.

#### 2.4.3. House Ventilation Calculation

Minute-by-minute data files were utilized to calculate the ventilation rates for each fan using the developed fan model. For intervals during which a fan was not operational for the full 60 s due to startup or shutdown, the calculated ventilation rates for those minutes were adjusted by the percentages of operational time. The total ventilation rate for the house at any given minute was determined by summing the rates from all 46 fans using Equation (3). These minute-by-minute ventilation rates were then averaged to obtain hourly or daily mean house ventilation rates for further analysis.

(3) $$V_{H,t}\;=\;\;\sum_{i=1}^{46}V_{Fi,t}$$

where *V_H,t_* is the ventilation rate of the entire house at a specific time (date, hour, and minute), m^3^ min^−1^; *V_Fi,t_* is the ventilation rate of the *i*th fan at the specific time, m^3^ min^−1^, obtained by using the fan model and multiplying by the percentage of time the fan is operating during that minute.

## 3. Results and Discussion

### 3.1. Differential Pressure

#### 3.1.1. Mean, Range, and Frequency of Differential Pressure

Over the six-month study, the mean house dP (average of the dP across the west and east walls) was −18.1 ± 8.9 Pa (mean ± standard deviation). The house experienced substantial fluctuations in dP, as recorded in AirDAC’s 1 min data files, ranging from +10.4 to <−100.0 Pa (Table 3). For a total of about 15 min during this study, the dP exceeded the ±100 Pa measurement range of the dP transducers. However, the dP remained between −10 and −30 Pa for 75.7% of this study’s duration. Positive dP, occurring 0.167% of the time, was only detected on the leeward side wall at an average wind speed of >24.5 km h^−1^. An extremely negative dP of <−50 Pa was observed for 0.435% of the time.

The literature reports that the means of dP in commercial manure belt laying hen houses were higher and the dP ranges were smaller than those in this study. There were only two studies that reported both means and ranges of dP. In the first 2-year study in the U.S., the means were 36.5 Pa (all Pa in this paragraph is absolute values) in one house and 48.9 Pa in another house, both houses with a range from 0 to >75 Pa [8]. In another 16-month study in Spain, the mean was 26.8 Pa with a range of 7.1–40.0 Pa [28]. Three more studies in the literature only reported dP ranges of 0–49 Pa [32], 2.7–35.4 Pa [33], and 0–52 Pa [34].

The literature reveals that, although dP in animal buildings is critical to ventilation, there are still limited data available. Various factors might affect the reported values, including the different methodologies applied in the studies, particularly the temporal resolution of the data. Because of the highly dynamic nature of wind velocities, lower temporal resolutions, such as 10 min or 1 hour, cannot capture rapid changes in dP.

#### 3.1.2. Effect of Wind on Differential Pressure

Wind direction and speed could drastically influence dP differences between the two sidewalls, regardless of the number of fans in operation. The prevailing winds across the area of this study were from the NW, W, or SW. The largest recorded dP difference between the two sidewalls over 1 min was 99.1 Pa, under a westerly (270°) strong breeze of 64.1 km h^−1^ on the morning of 26 March 2021. At that moment, the ventilation rates and fan energy efficiencies of the west wall fans greatly dropped.

**Figure 2:**
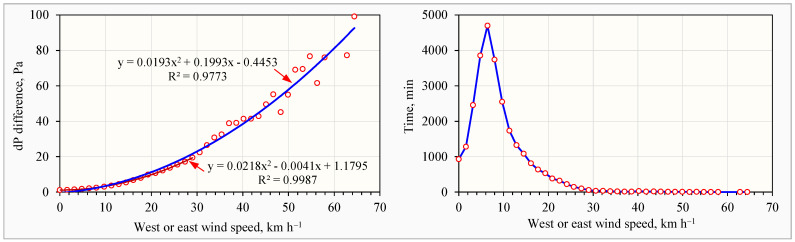
Wind speeds and differential pressures (dPs) during the six-month study. (**Left**) absolute dP differences between the west wall and east wall compared with 39 west (270°) or east (90°) wind speeds (at 1.6 km h^−1^ increment) averaged from 27,135 1 min data, selected from 265,405 min total valid data; (**right**) frequency of the recorded 27,135 1 min west or east winds at various speeds.

Westerly and easterly (90°) winds generated the highest negative dP across the house’s west and east walls, respectively. Analysis of the data recorded at these wind directions showed a strong correlation between wind speeds and dP. A second order polynomial equation with an R^2^ value of 0.9773 described this relationship for wind speeds up to 64.6 km h^−1^ (Figure 2, left). For wind speeds below 30.0 km h^−1^, a model with an R^2^ value of 0.9987 was more accurate. Westerly and easterly wind speeds exceeding 30.0 km h^−1^ were rare, occurring only 0.08% of the time (Figure 2, right).

#### 3.1.3. Effect of Fan Operation and Inlet Opening on Differential Pressure

**Figure 3:**
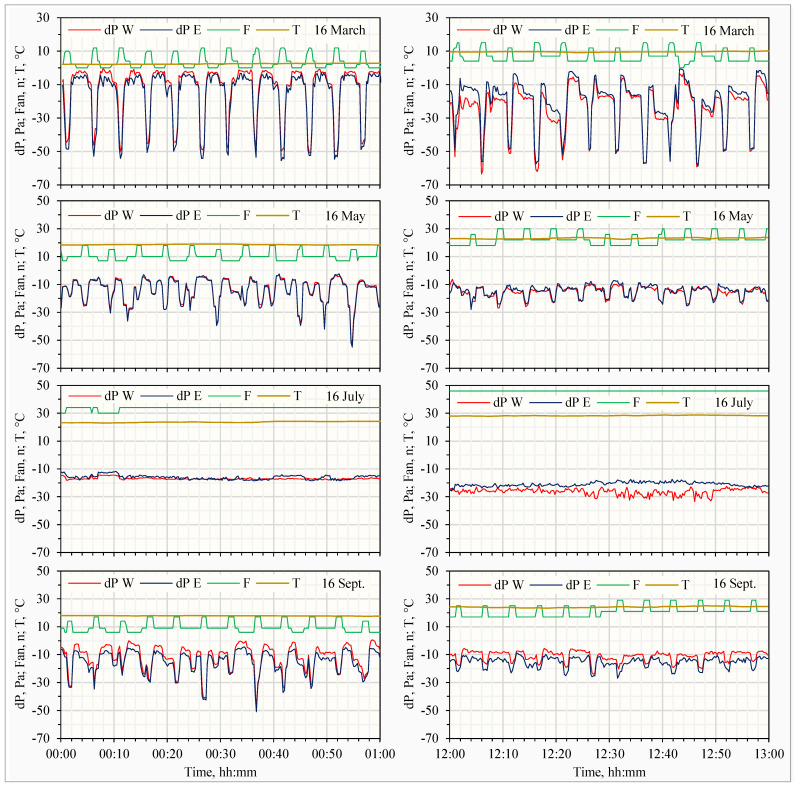
Examples of differential pressures (dPs) across the west wall (dP W) and east wall (dP E), number of fans operating in both walls (F), and outdoor temperature (T) on 4 selected days in various months using the 15 s data. One hour from midnight (**left charts**). One hour from noon (**right charts**).

The greatest variations in dP affected by fan operations occurred during intermittent operation of the eight cycling fans in stages 9 and 10 (Table 1), operating roughly one minute every five minutes. The start of these fans suddenly added a large proportion of fans in operation, as shown on the example days in March, May, and September in Figure 3. The fans achieved full rotational speeds within seconds after being turned on. However, the house baffle air inlet opening responded more slowly, or was not controlled to respond, causing sudden changes in the dP.

When the cycling fans operated continuously and the house air inlets were fully open in hot weather, fan operations were the sole factor affecting absolute dP, which varied more smoothly, as demonstrated in the example on 16 July (Figure 3). The increase in the number of operating fans from 30 or 34 at midnight to 46 at noon changed the house dP from −16.5 Pa to −24.0 Pa.

#### 3.1.4. Impact of Differential Pressure on Fan Efficiency and Ventilation Measurement

Differential pressures determine the efficiency of ventilation fans. As absolute dP rises from 12.4 Pa to 37.3 Pa and further to 74.4 Pa, fan ventilation rates can drop to 84.4% and 26.4%, and the fan ventilation efficiency may decrease to 77.7% and 21.8%, respectively (Table 2). Analysis of dP distribution revealed that the house was under dP of >−15 Pa and ≤−15 Pa for 40.2% and 59.8% of the time, respectively (Table 3). The house was under dP of ≤−40 Pa for 6.6% of the time.

As a comparison with the mean house dP of −18.1 ± 8.9 Pa in this study, a previous 16-month study reported a mean dP of 26.8 Pa [28]. Another study in two laying hen houses of similar design as in this study reported much higher mean house dPs of −36.5 Pa in one house and −48.9 Pa in another [8]. The results from both studies implied lower fan energy efficiencies. Therefore, in designing and managing mechanically ventilated animal housing, increasing ventilation energy efficiency can be considered alongside achieving relatively uniform air distribution.

Differential pressures can critically influence air distribution, which is crucial for achieving uniformity in the animal zone [7]. This uniformity primarily depends on the location and size of the air inlet [35]. To optimize the mixing of cold outdoor air with warm inside air during cold months, fresh air must be distributed without chilling the animals with drafts [36]. If inlet air velocities are too low during winter, because of inadequate dP in negative-pressure building systems, the denser cold air quickly descends to the animal level, adversely affecting animal health and performance [37].

Rapid fluctuations in dP, as shown in (Figure 3), also has a potential impact on fan ventilation characterization when using the traverse method [26] or the FANS [15]. Both methods involve traversing anemometer(s) through a 2-dimensional fan inlet or exhaust area and usually require from 45 min to about 1 hour for traverse [14] or at least a few min (e.g., the FANS required ~3 min) to complete one measurement. Variations in dP during this period can lead to inconsistent fan ventilation rate profiles and introduce significant errors if not accounted for.

### 3.2. Fan Rotational Speeds

**Figure 4:**
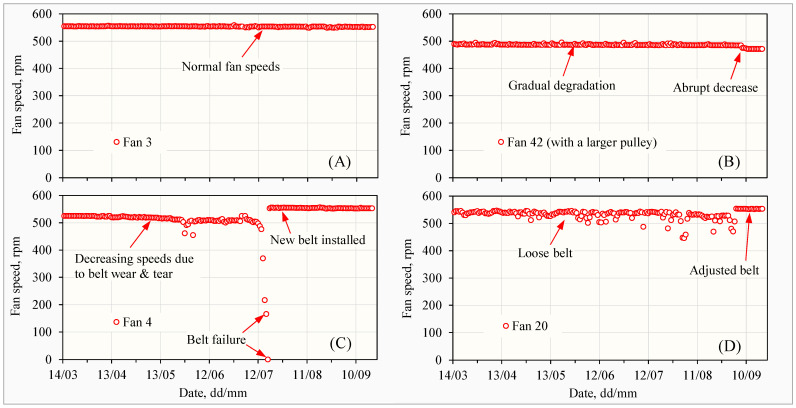
Example of four fans showing the maximum full fan rotational speeds each day during the six-month study. (**A**) At normal operation. (**B**) Affected by pulley size, gradual degradation, and abrupt change. (**C**) Affected by belt wear and tear, leading to belt failure. (**D**) Affected by loose belt.

Although they were single-speed fans, the full rotational speeds of the 46 fans under normal operating conditions ranged from 495 to 580 rpm, with an average of 555 ± 14 rpm. Well-maintained fans displayed stable performance with consistent speeds (Figure 4A). Further analysis showed that a fan with the lowest recorded speed of 495 rpm had a pully with a different diameter to the others (Figure 4B). In laying hen houses, pullies with different diameters could be purposely used for the same types of fans as reported [8], but could also occur for other unknown reasons, as shown in this study. Moreover, fan belt maintenance could substantially affect rotational speeds. Belt wear and tear could lead to belt failure (Figure 4C), and loose belts (Figure 4D) could result in decreased and/or unstable speeds, reducing ventilation efficiency.

Fan laws indicate that air volume varies directly with fan rotational speed [38]. The rotational speeds of belt-driven fans are influenced by factors such as power supply, dP, fan configuration, and fan maintenance. Fan power voltages can significantly impact airflow rates [9], and voltage fluctuations of 3.8% have been observed on commercial poultry farms [39]. Fan power voltage change is directly reflected in fan rotational speeds. Therefore, for the purpose of fan ventilation determination, it is not necessary to measure voltages if fan rotational speeds are measured. Differential pressures may also influence fan rotational speeds (Table 2).

The dynamics of fan rotational speeds, as shown in Figure 4, provide evidence and new understanding about the performance of ventilation fans under field conditions. The inclusion of continuously measured fan rotational speeds in the ventilation modeling can greatly improve the accuracies of calculated fan ventilation rates.

Based on the author’s field observations, the fan maintenance in this house was among the top levels compared with other egg layer farms. Throughout this study, findings on fan issues were communicated to the laying hen house manager and staff for timely maintenance, demonstrating how fan speed monitoring could be used as an early warning for quality ventilation management.

### 3.3. Wall Fan Ventilation Modeling

#### 3.3.1. Operation of the PFT

**Figure 5:**
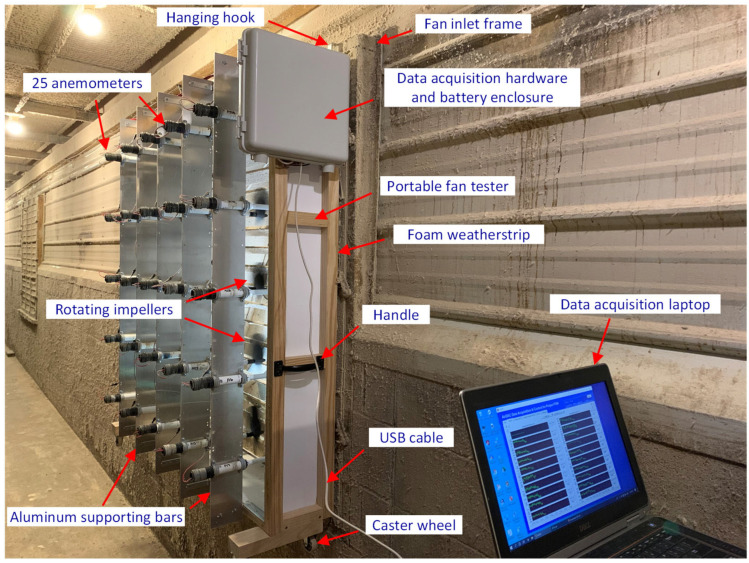
The full-size portable fan tester for measuring ventilation rates of a wall fan.

The PFT was found to be straightforward and practical for measuring wall fan ventilation. Weighing only 23 kg, including its data acquisition hardware and battery enclosure, the PFT was easily transported and set up at various fan locations within the laying hen house. The battery-powered design eliminated the need for power cords, enhancing operational convenience and safety (Figure 5). Transitioning from testing one fan to another required just about a couple of minutes.

**Figure 6:**
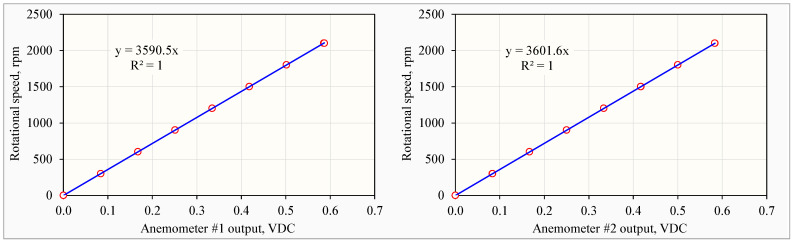
Example of calibration results of two anemometers at nine different rotational speeds, showing their slightly different *K* values of 3590.5 (**left**) and 3601.6 (**right**) for Equation (2).

The anemometers, individually calibrated and integrated into the PFT to reduce measurement errors (Figure 6), were capable of measuring air velocities with a precision of within 2% [30] and effectively accounted for the impacts of wind, dP, and fan speed on the measurements. The PFT system could generate measurement data continuously at 1 Hz with instantaneous response to the fast-varying dP, accurately capturing the dynamic changes in fan ventilation rates.

#### 3.3.2. Valid Fan Test Dataset

After analyzing data from the on-site fan test, a fan test result dataset, containing 133 valid data subsets from 29 of the 46 tested fans were obtained. This dataset spanned a range of dP from −1.2 to −59.0 Pa and fan rotational speeds from 490 to 570 rpm, representing typical house ventilation conditions across different seasons for fan model development. The air speeds in the 25 fan inlet sub-areas also provided data for in-depth analysis of the fan airflow profile in subsequent studies.

#### 3.3.3. Fan Ventilation Models

Fan ventilation rates in laying hen houses have high correlations with the house dP. Therefore, previously published ventilation fan models for laying hen houses were all based on house dP, e.g., [28,29]. Such a model developed in this study, as shown in Equation (4), had a coefficient of determination (R^2^) of 0.7114.

**Figure 7:**
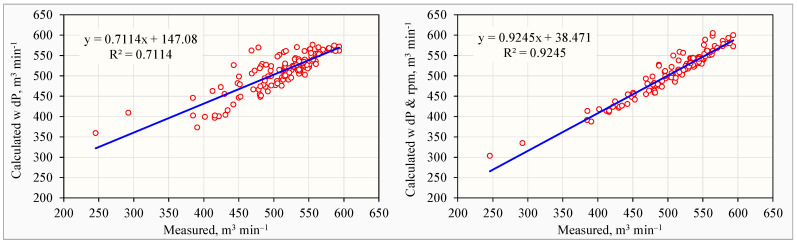
Comparison of the PFT-measured and model-calculated ventilation rates with Equation (4) based on dP from −1.2 to −59.0 Pa only (**left**) and with Equaiton (5) based on both dP from −1.2 to −59.0 Pa and fan rotational speeds from 490 to 570 rpm (**right**).

However, because of the fan laws [38] and the varying fan operation status in commercial laying hen houses, the inclusion of continuously measured fan rotational speeds in the fan model, as shown in Equation (5), considerably increased the model R^2^ value to 0.9245. Graphical comparison of the PFT-measured results with the model-calculated results clearly demonstrated the difference between the two models (Figure 7).

(4) $$V_{Fi,t}=3.7566\times dP_{Fi,t}+580.4$$

(5) $$V_{Fi,t}=-227.32+3.5616\times dP_{Fi,t}+1.4779\times R_{Fi,t}$$

where *dP_Fi,t_* is the differential pressure across the wall where installed *i*th fan at time *t*, Pa; *R_Fi,t_* is the rotational speed of the *i*th fan at time *t*, rpm.

### 3.4. House Ventilation Rate

#### 3.4.1. House Ventilation Rate at Different Time Scale

By using Equations (5) and then (3) and averaging the 1 min results, the calculated hourly mean house ventilation rates over the 6-month study ranged from 1342 to 22,436 m^3^ min^−1^ (Table 4). The average hourly mean ventilation rate ± standard deviation (AHM ± Std) was 10,833 ± 7074 m^3^ min^−1^, and the AHM ± confidence interval (c.i.) was 10,833 ± 210 m^3^ min^−1^. The daily mean house ventilation rates ranged from 1800 to 22,142 m^3^ min^−1^, and the average daily mean (ADM) house ventilation rate was 10,803 ± 5906 m^3^ min^−1^ (ADM ± Std). The ADM ± c.i. house ventilation rate was 10,803 ± 860 m^3^ min^−1^. The ADM ventilation rate per bird was 0.078 ± 0.043 m^3^ min^−1^ or 4.68 ± 2.58 m^3^ h^−1^.

#### 3.4.2. Outdoor Temperature and House Ventilation Rate

On colder days, the west wall fans (fans 1–23) generated about 10% more ventilation than the east wall fans (fans 24–46) because of the configuration of fan stages. The two stage 1 fans (fans 4 and 19, Table 1) were both on the west wall. Consequently, when only one stage of fans was in operation, no fans on the east wall were running. This might cause an uneven distribution of fresh air between the west and east sides of the house.

**Figure 8:**
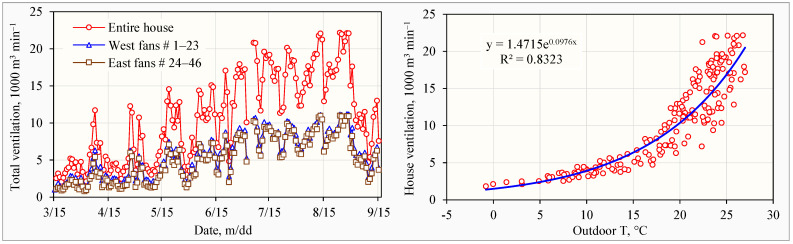
Daily mean house ventilation rates (**left**) compared with outdoor temperatures (**right**) over the six-month study.

Throughout this study, daily mean house ventilation rates increased from mid-March to early September, then decreased until the end of the study period (Figure 8, left). The decrease coincided with the conclusion of artificial molting on September 2 and could be related to changes in house ventilation control setting. Nevertheless, the dynamics in house ventilation from cold to hot seasons were highly correlated with variations in outdoor temperatures and can be modeled with an exponential function (Figure 8, right). A model with the same form (y = 0.7504e^0.1146x^; where y is in 10^6^ m^3^ d^−1^; x is outdoor T, ranging from 3.5 to 26.5 °C) for a 52,000-hen enriched-cage manure belt laying hen house in the Basque Country, Spain was obtained by Alberdi et al. [29]. This demonstrated similar automatic ventilation control strategies for laying hen houses in the two countries.

#### 3.4.3. Ventilation Monitoring Data Completeness

This 6-month study collected 4416 h, or 184 days, of total measurement data thanks to the adoption of a comprehensive measurement approach and high-level quality assurance and quality control measures. Using the criteria for “valid data hours” and “valid data days”, which only include hours and days with at least 75% valid data (45 min per hour and 18 h per day) to minimize bias [24], resulted in 4379 valid hours and 181 valid days. The data completeness for hourly and daily averages was 99.2% and 98.4%, respectively, marking this study as one of the highest in terms of data completeness among long-term studies monitoring ventilation rates in large animal buildings. Continuous fan rotational speed and building dP measurement combined with on-site fan testing and fan model calculation demonstrated reliability for long-term and high-quality ventilation monitoring at the large commercial laying hen house.

#### 3.4.4. Ventilation Rate and Monitoring Technique

Over the past 20 years, only approximately a dozen studies have reported ventilation measurement results in commercial manure belt laying hen houses across Canada, China, Italy, Spain, and the U.S. (Table 5). These results varied greatly, ranging from 0.43 to 17.0 m^3^ h^−1^ hen^−1^. Some of these variations reflected seasonal differences, with higher ventilation rates in the summer and lower rates in the winter [40,41,42]. For instance, in two U.S. houses, the monthly mean ventilation rates were 4.87 and 5.01 m^3^ h^−1^ hen^−1^ in July, and 0.59 and 0.81 m^3^ h^−1^ hen^−1^ in February, respectively [8].

Methodologies and technologies used in ventilation measurements were also critical factors behind these variations. Time coverage (including both day and night times, and different seasons) and measurement duration (from one day to two years, Table 5) are essential due to the diel and seasonal ventilation variations and the highly dynamic ventilation rates in laying hen houses. Ventilation rates can vary significantly among different days, even within the same season (Figure 8, left). Bias can easily be introduced if the time coverage and measurement duration are not adequately planned.

Monitoring ventilation in animal buildings involves capturing relevant signals and using mathematical models to convert these signals into ventilation rates. In mechanically ventilated buildings, there are more than a dozen possible signals, ranging from “upstream” fan power supply to “downstream” airflow from the fans [46]. Among these signals, “downstream” fan ventilation airflow is recognized as the most reliable. Therefore, most studies in Table 5 adopted either FANS or an anemometer to measure fan airflows and then used mathematical models to calculate ventilation rates. The PFT in this study improved these methods by enabling a quick response to capture sufficient ventilation dynamics under varying dP and fan rotational speeds. However, as a traverse-based method, the PFT is intrusive and can introduce some errors like other similar methods. Theoretically, the ventilation rate measured with traverse methods can be slightly lower than the true ventilation rate.

FANS, anemometers, and PFT can only perform sporadic fan tests. Longer-term measurements of dP, fan status (on/off or rotational speeds), or carbon dioxide (CO_2_) concentration were applied to calculate ventilation rates using mathematical models. The measurement results in Table 5 were all estimates of the true values and were imperfect [34]. Nonetheless, the relatively higher reliability of these results can be assessed based on the methodologies, technologies, and quality control in the measurements.

## 4. Conclusions

The house experienced significant variations in differential pressure (dP) during this study. House dP was greatly influenced by wind velocities. Extremely high and positive dP values were induced by strong breezes. The number of operating fans and the opening of air inlets directly affected the house dP. Intermittent operation of the cycling fans, combined with the slow response of house air inlet baffles, led to sudden changes in dP. Rotational speeds of single-speed fans under field conditions can vary. Pulley diameters, belt wear and tear, and belt tightness affected fan speeds. Fan speed monitoring can be used to improve ventilation fan management. House ventilation rates were highly dynamic and strongly correlated with outdoor temperatures and hen molting. The daily mean ventilation rates averaged 4.68 m^3^ h^−1^ hen^−1^ in this study. Continuous fan rotational speed and house dP measurements, combined with on-site fan testing and ventilation rate modeling, proved reliable for long-term and high-quality ventilation monitoring in the large commercial laying hen house. On-site fan testing with a fast-response fan tester and the inclusion of both the continuously measured dP and fan rotational speed in the ventilation modeling considerably improved the accuracy of ventilation calculations.

## Figures and Tables

**Table 1:** Fan stages and fan numbers in the laying hen house.

Fan Stage	Fan Number						Sidewall
1	4	19					West
2	27	42					East
3	1	12	23				West
4	24	35	46				East
5	2	8	15	21			West
6	25	31	38	44			East
7	6	9	14	17			West
8	29	32	37	40			East
9	3	11	13	20			West (cycling)
10	26	34	36	43			East (cycling)
11	5	7	10	16	18	22	West
12	28	30	33	39	41	45	East

**Table 2:** Characteristics of a RayDot PMC-4821BD fan in BESS lab test #97323 [21].

Differential Pressure, Pa	Speed, rpm	Ventilation, m^3^ min^−1^	Efficiency, m^3^ W^−1^
0.0	591	696.2	42.6
12.4	589	653.7	38.1
24.9	587	602.8	33.1
37.3	586	551.9	29.6
49.8	586	478.3	24.8
62.2	584	345.3	17.5
74.7	582	172.6	8.3

**Table 3:** Frequency of differences between the differential pressures (dPs) across west and east walls in the laying hen house from March 15 to September 15 using 1 min data.

dP Range, Pa	West Wall		East Wall		Average Time, %
	Time, min	Time, %	Time, min	Time, %	
>10	–	0	62	0.023	0.012
5 to 0	161	0.061	663	0.250	0.155
0 to −5	14,055	5.30	16,514	6.22	5.76
−5 to −10	29,975	11.3	32,389	12.2	11.7
−10 to −15	56,763	21.4	63,002	23.7	22.6
−15 to −20	53,903	20.3	51,895	19.6	19.9
−20 to −25	56,502	21.3	39,069	14.7	18.0
−25 to −30	34,363	12.9	46,453	17.5	15.2
−30 to −35	8439	3.18	6226	2.35	2.76
−35 to −40	4899	1.85	4072	1.53	1.69
−40 to −45	3166	1.19	2575	0.970	1.08
−45 to −50	1814	0.683	1544	0.582	0.633
−50 to −55	770	0.290	611	0.230	0.260
−55 to −60	283	0.107	179	0.067	0.087
−60 to −65	167	0.063	63	0.024	0.043
<−65	149	0.056	88	0.033	0.045

**Table 4:** Ranges, average hourly mean (AHM), and average daily mean (ADM) of the house ventilation rates over the six-month study.

Statistics	Ventilation Rate, m^3^ min^−1^		
	Fan 1–23	Fan 24–46	House (Fan 1–46)
Hourly mean range	612–11,616	489–11,663	1342–22,436
AHM ± Std	5687 ± 3529	5178 ± 3580	10,833 ± 7074
AHM ± 95% c.i.	5687 ± 105	5178 ± 106	10,833 ± 210
Daily mean range	1032–11,192	768–10,987	1800–22,142
ADM ± Std	5676 ± 2941	5178 ± 2976	10,803 ± 5906
ADM ± 95% c.i.	5676 ± 429	5178 ± 430	10,803 ± 860

**Table 5:** Comparison of reported ventilation per hen (V_hen_) and measurement techniques in commercial manure belt laying hen houses with mechanical ventilation.

House Size, Duration, and Location ^(a)^	V_hen_, m^3^ h^−1^ hen^−1 (b)^	Technique ^(c)^	Report Year
98,000 hens, 1 d (31 December), Iowa, U.S.	0.43	FANS, CO_2_ balance	2005 [32]
100,000 hens, same house, 1 d (22 July), Iowa, U.S.	5.28	FANS, CO_2_ balance	2005 [32]
169,000 hens, 176 d (August–January), Ohio, U.S.	2.78 (0.56–7.26)	FANS, dP, fan relay on/off	2006 [33]
60,000 hens, 1 week × 6 periods over a year, Northern Italy	6.2 (3.0–17.0)	Vane, fan speed, CO_2_ balance	2007 [43]
250,000 hens, house A, 693 d (2 years), Indiana, U.S.	2.08 (0.59–4.87)	FANS, dP, fan on/off, and fan speed ^(2)^	2012 [8]
250,000 hens, house B, 678 d (2 years), Indiana, U.S.	2.10 (0.81–5.01)	FANS, dP, fan on/off, and fan speed ^(2)^	2012 [8]
22,000 hens, 1 year, Northern Italy	0.8	HWA, fan on/off	2012 [41]
November–May	0.62		
June–November	1.01		
67,500 hens, 91 d in 4 seasons, Ontario, Canada ^(1)^	(0.44–8.44)	FANS, no other technique reported	2014 [44]
52,000 hens, 18 mo (12 April–13 September), Basque Country, Spain	0.88–13.30	HWA, dP, fan on/off	2016 [29]
39,000 hens, 16 mo (15 July–16 October), Basque Country, Spain		Three different methods	2019 [28]
	5.3 ± 2.9 (1.1–11.6)	HWA	
	5.9 ± 3.3 (1.1–13.1)	Fan speed	
	6.3 ± 2.1 (2.8–10.7)	CO_2_ balance	
100,000 hens, 15 d, Beijing, China		CO_2_ balance	2020 [42]
6 d in August	3.79 ± 1.44		
6 d in October	0.95 ± 0.45		
3 d in January	0.54 ± 0.25		
140,000 hens, 53 d (July–October), Midwestern U.S.	4.0 ± 0.4 (0.8–9.1)	FANS, fan relay on/off	2020 [34]
170,000 hens, House 1, 1 year, Ohio, U.S.	1.83	FANS, dP, fan on/off	2021 [45]
170,000 hens, House 2, 1 year, Ohio, U.S.	1.74	FANS, dP, fan on/off	2021 [45]
140,000 hens, 181 d (February–September), Midwestern U.S. (this study)	4.68 ± 2.58 (0.77–9.74)	PFT, dP, fan speed	2024

Note: ^(a)^ The numbers are the reported approximate numbers of hens in the house. ^(b)^ The numbers in parentheses are V_hen_ ranges. ^(c)^ FANS—fan assessment numeration system [32]; vane—vane anemometer; HWA—hot wire anemometer; PFT—portable fan tester. ^(1)^ 91 d in 4 seasons: fall 39 d, winter 29 d, spring 15 d, summer 34 d. The V_hen_ range is estimated from Figure 1 in [44]. ^(2)^ Fan on/off monitoring on single-speed fans, and fan rotational speed monitoring on variable-speed fans.

## Data Availability

The data that support the findings of this study are available from the corresponding author upon reasonable request.
